# Generalized redshift formula through an energy-based framework

**DOI:** 10.1038/s41598-024-73191-4

**Published:** 2024-09-30

**Authors:** C. Ortiz, F. Ibarra-Castor

**Affiliations:** Unidad Académica de Física, Universidad Autónoma de Zacatecas, Zacatecas, 98060 México

**Keywords:** Cosmology, Astronomy and astrophysics, Physics

## Abstract

Redshift is a crucial concept in physics; it has significant implications for our understanding of the dynamics and evolution of the cosmos. In this article, we introduce a generalized formula to determine the redshift parameter. The unified framework, which relates the redshift to the energy of the system, eliminates the need to derive the redshift parameter on a case-by-case basis, uncovering the relationships between different mechanisms. Furthermore, the generalization allows us to extend the redshift to non-considered mechanisms.

## Introduction

In 1842, the Austrian physicist Christian Doppler introduced a groundbreaking concept, now known as the Doppler effect^1^. He articulated a fundamental principle that illustrates that the observed frequency of a wave can be altered by the relative motion between the observer and the source of the wave. This relative motion plays a crucial role in influencing the measured frequency when the wave propagates through a specific medium. Doppler’s pioneering work laid the foundation for understanding the lengthening of photon’s electromagnetic radiation, today known as redshift. The electromagnetic redshift is attributed to three mechanisms. One is the *Doppler shift*, which occurs when radiation is emitted from a moving source relative to the observer. Another cause is the *gravitational redshift*, which arises when radiation moves away from an object, towards a weaker gravitational potential. Lastly, the *cosmological redshift* occurs as the radiation travels through expanding space. The redshift formulae are obtained independently for all cases.

There have been some attempts to generalize the concept of redshift. The mathematician Hermann Weyl^2^ provided a general definition of the frequency shift of light in arbitrary spacetime. He related the redshift to the line element, $\textrm{ds}$, for null geodesics, as $\mathrm {1+z=ds_\textrm{o}/ds_\textrm{e}}$. The idea was further refined by Harvey et al.^3^ by making use of the concept of killing-vector fields. In recent literature, there are several articles that aim to extend and generalize the determination of redshift^4,5^, as well as its application and detection in gravitational waves^6,7^.

It is worth noting that its derivation is complicated and that non-conservative mechanisms are not considered in these formulations.

In this article, we introduce a unified formula to determine the redshift of waves or particles, irrespective of the underlying mechanisms driving the redshift. Our approach to generalizing the redshift is grounded in the principle of energy conservation. The derived generalized redshift formula simplifies both the calculation and interpretation of the redshift, making it applicable across diverse physical domains. This holds promise for a broad spectrum of applications in various scientific disciplines. Following the formulation of the generalized redshift equation, we proceed to employ it to derive the principal known redshift mechanisms.

## Redshift

Before delving into the concept of redshift, it is essential to understand the Doppler effect. This effect occurs when there is relative motion between an observer and the source of a wave, resulting in a noticeable shift in the frequency of the wave. Its mathematical equation is the ratio between the emitted frequency $f_\textrm{e}$ and the observed frequency $f_\textrm{o}$,

1 $$\begin{aligned} \frac{f_\textrm{e}}{f_o}=\frac{c_\textrm{s}\pm \textrm{v}_\textrm{e}}{c_\textrm{s}\pm \textrm{v}_\textrm{o}}, \end{aligned}$$

where $c_s$ is the propagation speed of the waves in the medium $s$, $\textrm{v}_{\text {e}}$ is the speed of the emitter relative to the medium and $\displaystyle \textrm{v}_{\text {o}}$ is the speed of the observer relative to the medium. As can be seen, the ratio of frequencies is directly proportional to the ratio of the relative velocities between the observer and the emitter. The Doppler effect was extended to light waves by the French physicist Armand Fizeau in 1848^8^, who independently developed the principle and related the shift in the electromagnetic spectral lines of stars to the Doppler effect.

Today, the shift in electromagnetic spectral lines is known as redshift and is represented by the dimensionless quantity $z$. Defining the difference between observed and emitted wavelength as $\Delta \lambda = \lambda _\textrm{o} - \lambda _\textrm{e}$, redshift is associated with the ratio of observed and emitted wavelengths as $z=\Delta \lambda /\lambda _\textrm{e}$. Furthermore, considering the difference between observed and emitted frequency $\Delta f = f_\textrm{o} - f_\textrm{e}$, the redshift corresponds to the frequency ratio, $z=-\Delta f/f_\textrm{o}$.

Although redshift stands as a firmly established observational phenomenon, its interpretation varies depending on the mechanisms driving the phenomenon. Among the known redshift mechanisms, we emphasize the Doppler effect, gravitational effect, and space expansion. The preceding classification is not general enough; in the context of redshift, we introduce a novel equation that establishes a connection between redshift and energy. This equation offers a fresh perspective on quantifying and understanding the redshift.

## Redshift generalization

In order to formulate a generalized redshift equation, we need to distinguish between the observational phenomena and the physical mechanism that causes the effect. The formula must relate the observational parameters, wavelength/frequency, and the variables of the underlying mechanism. As discussed previously, the Doppler redshift is related to changes in coordinates, the gravitational redshift with a gravitational potential, and the cosmological redshift with modifications in the line element; all of them can be related to the energy. The wavelength and frequency of a waveform are related to the phase velocity $\textrm{v}$, by the linear relation $f \lambda = \textrm{v}$. Recalling the energy per unit mass, the specific energy $\mathscr {E}$, of a particle in the ground state, a proportionality relation can be established to the square of the phase velocity, $\mathscr {E}=\mathrm {v_{\gamma }}^2/2$. Assuming that the photon energy momentum is conserved, a general relation can be determined between these quantities and the square root of the specific energy, $\lambda ^{-1} \propto \textrm{v} \propto \mathscr {E}^{1/2}$.

Energy is a conserved quantity that is well understood in all fields of physics. In the present work we introduce the redshift as minus the change of the square root of the specific energy over the observed/final specific energy $\mathscr {E}_\textrm{o}$,

2 $$\begin{aligned} z\equiv -\frac{\Delta (\mathscr {E}^{1/2})}{\mathscr {E}_{\textrm{o}}^{1/2}}. \end{aligned}$$

We must acknowledge that the redshift definition is given in terms of the specific energy since we are considering massless particles such as photons. The aforementioned restriction can be relaxed for conserved massive particles because the redshift is the ratio of the energy, and it does not depend on the mass of the particle.

The redshift formula can be recast as the ratio of the square root of the initial emitted specific energy of the system, over the square root of the observed specific energy, which is a more useful redshift relation,

3 $$\begin{aligned} 1+z\equiv \frac{ \mathscr {E}_\textrm{e}^{1/2}}{\mathscr {E}_{\textrm{o}}^{1/2}}. \end{aligned}$$

We will refer to the equation mentioned above as the “redshift relation”. The aforementioned relation has the advantage of being a ratio of an initial state to the final or observed one. Depending on the conserved quantities, this relation gives the velocity ratio, energy ratio, the line element ratio, or expansion ratio between the emitter and the observer.

The generalized redshift formula allows us to derive the observational redshift, as well as all known redshift mechanisms. To derive observational redshift, we need to recast the generalized redshift formula in terms of the observed variable, frequency, or wavelength. It is important to consider that, in most cases, only the observed energy can be known with certainty; therefore, in many cases we may not be able to distinguish which is the underlying mechanism causing the redshift.

To have consistent measurements, the initial energy must correspond to the expectation value of the Hamiltonian of the system in the ground state; this is the zero-point energy of the system $E=h f/2$^9,10^. Using the generalized redshift formula by casting the specific energy in terms of frequency $\mathscr {E}={h^2 f}^2/2p^2$ and considering that the momentum of the photon is conserved, the redshift can be recast in terms of frequency,

4 $$\begin{aligned} z=\frac{f_\textrm{e}-f_\textrm{o}}{f_\textrm{o}}. \end{aligned}$$

Redshift mechanisms refer to the physical processes or phenomena that cause the observed redshift in the spectra of objects under observation. The generalized formula allowed us to quantify the redshift, which can be classified into two categories depending on the nature of energy loss: one that involves changes in coordinates and another due to changes in potential. If the emitter is in a moving reference frame or the system is subject to nonholonomic constraints, then a change of coordinates must be performed to the kinetic energy term of the generalized formula. When the change of energy depends only on the position and holonomic constraints, the final energy may contain the kinetic term in the rest frame plus the adequate potential. We can further extend the applicability of the redshift relation (3), in order to compare the energy between two non-ground-state systems; the resulting relation is the quotient of the involved redshift relations.

In the following, using the redshift generalization formula and a unified interpretation, we derive the redshift due to the well-known mechanisms.

## Doppler redshift

The Doppler effect^1^ refers to the change in frequency (and thus wavelength) of a wave perceived by an observer as a result of the relative motion between the source of the wave and the observer. It can be applied to both sound and light waves.

In 1868, Huggins^11^ was the first to associate velocity with the shift of spectral lines. It was until 1900 that Bélopolsky^12^ made a direct demonstration of the Doppler-Fizeau principle in a laboratory.

The Doppler redshift mechanism is a fundamental concept in physics and plays a central role in various astrophysical studies. When an object moves away from an observer, the light it emits undergoes a change in wavelength relative to the observer, resulting in a redshift towards the longer wavelengths of the spectrum. This redshift is a consequence of the differing reference frames between the observer and the emitter, and does not involve any actual gain or loss in the energy of the photon. This phenomenon has significant implications for our understanding of celestial motion and the expanding universe. In the field of astronomy, the Doppler redshift is a crucial tool for analyzing the velocities of distant celestial objects, such as galaxies and stars. By exploring the redshift of light from these sources, scientists can unlock valuable insights into the dynamics and evolution of the cosmos.

Using the photon momentum-wavelength relation along with the phase-velocity relation, we can express the specific kinetic energy in relation to the photon’s phase velocity as $\mathscr {E}=\mathrm {v_{\gamma }}^2/2$. The redshift or displacement of the photon’s wavelength resulting from a moving reference frame can be derived using the generalized redshift formula (2). The initial specific energy is given by the specific kinetic energy of the emitted photon in a moving reference frame, and subject to a time dilation due to relativistic corrections,

5 $$\begin{aligned} \mathscr {E}_\textrm{e}= \frac{1}{2}\left( \frac{\textrm{d}r_\textrm{e}}{\textrm{d}t}\right) ^2, \end{aligned}$$

and the final specific energy, in terms of the velocity of the observed particle,

6 $$\begin{aligned} \mathscr {E}_o= \frac{1}{2}\left( \frac{\textrm{d}r}{\textrm{d}t}\right) ^2. \end{aligned}$$

As the redshift mechanism depends on a moving frame of reference, the change of energy is in the kinetic term. In such cases, to have the system in the same frame of reference, a transformation of coordinates is needed. Consequently, the Doppler redshift for a source that is displacing away from the observer at a velocity $\textrm{v}_{\Vert }$ is given by

7 $$\begin{aligned} z=\frac{\sqrt{\frac{1}{2}\left( \frac{\textrm{d}r_\textrm{e}}{\textrm{d}t}\right) ^2}-\sqrt{\frac{1}{2}\left( \frac{\textrm{d}r}{\textrm{d}t}\right) ^2} }{\sqrt{\frac{1}{2}\left( \frac{\textrm{d}r}{\textrm{d}t}\right) ^2}}, \end{aligned}$$

where $\textrm{d}r_\textrm{e}= \textrm{d}r+\textrm{v}_{\Vert }\textrm{d}t$ is transformation of coordinates of the moving reference frame. The dilation of time is mathematically expressed by the relation $\textrm{d}t'=\textrm{d}t/\sqrt{1-\textrm{v}^2/c^2}$,

8 $$\begin{aligned} z=\frac{\sqrt{\frac{1}{2}\left( \frac{\textrm{d}r+\textrm{v}_{\Vert }\textrm{d}t}{\textrm{d}t/\sqrt{1-\textrm{v}^2/c^2}}\right) ^2}}{\sqrt{\frac{1}{2}\left( \frac{\textrm{d}r}{\textrm{d}t}\right) ^2} }-1. \end{aligned}$$

For a particle traveling at the speed of light $\textrm{d}r/\textrm{d}t=c$,

9 $$\begin{aligned} 1+ z=\frac{1+\frac{\textrm{v}_{\Vert }}{c}}{\sqrt{1-\frac{\textrm{v}^2}{c^2}}}. \end{aligned}$$

If the reference frame moves in the radial direction $\textrm{v}_{\Vert }= \textrm{v}$,

10 $$\begin{aligned} 1+ z_{\Vert }=\frac{ \sqrt{1+\frac{\textrm{v}}{c}}}{\sqrt{1-\frac{\textrm{v}}{c}}}. \end{aligned}$$

In the classical limit, $\textrm{v}\ll c$, the redshift equation is recast as:

11 $$\begin{aligned} z_{\Vert }\approx \frac{\textrm{v}}{c}. \end{aligned}$$

As for the motion in the transverse direction, $\textrm{v}_\Vert =0$,

12 $$\begin{aligned} 1+ z=\frac{1}{\sqrt{1-\frac{\textrm{v}^2}{c^2}}}, \end{aligned}$$

in the classical limit, the redshift manifests as,

13 $$\begin{aligned} z_{\perp }\approx \frac{1}{2}\left( \frac{\textrm{v}}{c}\right) ^2. \end{aligned}$$

The formula presented for the redshift generalization accurately predicts the Doppler redshift in the relativistic context and in its classical limit.

We must point out that the Doppler redshift effect is due to a difference in the frame of reference between the emitter and observer. There is no real energy loss of photon.

## Gravitational redshift

Gravitational redshift has been employed to study the properties of celestial objects, such as stars and black holes. It has also been used as a valuable tool in testing the predictions of Einstein’s theory of general relativity or beyond^13^. Understanding the gravitational redshift mechanism is essential for interpreting the observed spectra of distant objects and gaining deeper insights into the nature of gravity and the behavior of light in the presence of massive bodies.

The gravitational redshift mechanism arises because of the influence of gravity on light and other forms of electromagnetic radiation. As light travels through a region with a stronger gravitational field, its wavelength is stretched, leading to a redshift, which means the light shifts towards the lower-frequency, longer-wavelength end of the electromagnetic spectrum. This effect is one of Einstein’s classical tests predicted by the General Theory of Relativity^14–16^. Pound and Rebka^17^ proved it in 1959, showing that the equivalence principle leads to an equivalent Doppler shift of electromagnetic radiation.

The gravitational redshift can be derived by making use of the generalized redshift relation (3). We must consider the initial specific energy, which is represented by the kinetic energy in the rest frame. When the change in energy depends on the position, it is necessary to include the appropriate potential energy. Therefore, the final specific energy is given in terms of the initial kinetic energy and the gravitational potential.

14 $$\begin{aligned} \mathscr {E}_o= \frac{1}{2}\left( \frac{\textrm{d}r}{\textrm{d}t}\right) ^2 -\frac{GM}{r}. \end{aligned}$$

The redshift due to a gravitational potential is given by equation (2):

15 $$\begin{aligned} z=\frac{\sqrt{\frac{1}{2}\left( \frac{\textrm{d}r}{\textrm{d}t}\right) ^2 }-\sqrt{\frac{1}{2}\left( \frac{\textrm{d}r}{\textrm{d}t}\right) ^2 -\frac{GM}{r} }}{\sqrt{\frac{1}{2}\left( \frac{\textrm{d}r}{\textrm{d}t}\right) ^2 -\frac{GM}{r}}}. \end{aligned}$$

Considering that the speed of a photon is constant *c*, we arrive at the usual gravitational redshift relation,

16 $$\begin{aligned} 1+z=\frac{1}{\sqrt{1 - \frac{2GM}{rc^2} }}. \end{aligned}$$

In the Newtonian limit, $r\gg 2GM/c^2$,

17 $$\begin{aligned} z\approx \frac{2GM}{rc^2} =\frac{\Delta U}{c^2}, \end{aligned}$$

where $\Delta U$ is the gravitational potential. It is important to note that this is the same result obtained when considering the change in the line element of a particle resulting from a gravitational field.

The applicability of the redshift can be further extended in order to derive the redshift between light in arbitrary potentials. Since the redshift relation (3) is cast as a ratio, we can compare the redshift relation between the light in the ground state and the light in the emitter potential, $1+z_\textrm{e}$ with the redshift of the ground state and the light in the observers potential, $1+z_\textrm{o}$.

In the following, we calculate the redshift of an emitted light subject to a certain gravitational potential and observed at a different potential. First, we calculate the redshift between the light in the rest reference frame and the emitted light as Eq. (16),

18 $$\begin{aligned} 1+ z_\textrm{e}=\frac{1}{\sqrt{1 - \frac{2GM}{r_\textrm{e}c^2} }}. \end{aligned}$$

Then, we compare it with the redshift between the light in the rest reference frame and the light in the observer space-time reference frame,

19 $$\begin{aligned} 1+ z_\textrm{o}=\frac{1}{\sqrt{1 - \frac{2GM}{r_\textrm{o}c^2} }}. \end{aligned}$$

The redshift of a photon particle emitted at a given potential $U(r_\textrm{e})$ and observed at a different potential $U(r_\textrm{o})$ is given by the ratio of the respective redshifts,

20 $$\begin{aligned} 1+ z_{\textrm{e} \rightarrow \textrm{o} } =\frac{ 1+ z_\textrm{e} }{1+ z_\textrm{o} } =\frac{\sqrt{1 - \frac{2GM}{r_\textrm{o}} }}{\sqrt{1 - \frac{2GM}{r_\textrm{e}} }}. \end{aligned}$$

The result is the same as the one obtained by using the Weyl formulation, based on the line element. The advantage of the proposed redshift generalization lies in its interpretation in terms of energy and its wider applicability.

Contrary to Doppler redshift, gravitational redshift involves a actual change in energy due to a potential difference between the emitter and the receiver.

## Cosmological redshift

The cosmological redshift refers to the observed shift in wavelength, which is explained as a result of the apparent expansion of the Universe^18^. When light travels from distant galaxies to us, it passes through expanding space, causing its wavelengths to extend progressively. This elongation leads to an increase in wavelengths, which leads to the characteristic reddening of light. It is crucial to differentiate this occurrence from the Doppler effect, which emerges due to the inherent motion of the light source.

By critically examining these mechanisms, including their implications and limitations, we aim to contribute to the ongoing discourse surrounding the nature of the redshift phenomenon and its potential cosmological implications. We must mention that the cosmological redshift is currently the only redshift mechanism that has not been directly tested experimentally. However, it is theoretically possible to measure the change over time in the redshift between an emitter and an observer comoving with the Hubble flow^19–23^, known today as the redshift drift. There are several experimental proposals aimed at detecting this phenomenon^24,25^.

This phenomenon can be obtained by compering the kinetic energy at the present time with respect to the one at the emission time. Because we do not know whether the Universe is finite or infinite, it does not make sense to talk about an absolute size, or radius, of the Universe. Instead, we use an arbitrary, dimensionless scale called the scale factor, usually denoted $a(t)$. Since a homogeneous and isotropic expansion is assumed, we use the usual relation between the proper distance $\vec {r}$ and the comoving distance $\vec {x}$ given by the relation $\vec {r}=a(t)\vec {x}$, where the scale factor, $a(t)$, measures the rate of expansion of the Universe at a given time. The scale factor itself does not mean anything. The important thing is the ratios of the two scale factors.

The energy considered is given by the kinetic energy, which is the one that undergoes the effect of space expansion.

The redshift can be cast by making use of the redshift relation (2),

21 $$\begin{aligned} z=\frac{\sqrt{\frac{1}{2} (\frac{\textrm{d}r}{\textrm{d}t})^2|_{t_\textrm{e} }}-\sqrt{\frac{1}{2} (\frac{\textrm{d}r}{\textrm{d}t})^2|_{t_\textrm{o} }} }{\sqrt{\frac{1}{2} (\frac{\textrm{d}r}{\textrm{d}t})^2|_{t_\textrm{o} }}}. \end{aligned}$$

By recalling the equation of signal that propagates along a light-like expanding worldline $$\textrm{d}s^2=0=$$$$c^2\textrm{d}t^2-a(t)^2\textrm{d}r^2$$, the redshift is recast as,

22 $$\begin{aligned} 1+z=\frac{\frac{c\textrm{d} t}{ a(t_\textrm{e})\textrm{d}t} }{\frac{c\textrm{d}t}{ a(t_\textrm{o})\textrm{d}t}}=\frac{a(t_\textrm{o})}{a(t_\textrm{e})}. \end{aligned}$$

We must acknowledge that we are describing phenomena in the system of reference of the observer; hence, the scale factor at $t_\textrm{o}$ is normalized to unity, $a(t_\textrm{o}=0)=1$. By contrast, the scale factor at $t_\textrm{e}$, is the one on which the photon was emitted $a(t_\textrm{e})=a$, so the cosmological redshift relation is cast as:

23 $$\begin{aligned} 1+ z=\frac{1}{a}. \end{aligned}$$

By assuming a time parameterization of the distance, and using the geodesic equation of a light wave, the redshift generalization formula leads to the cosmological redshift. Through a generalized formula, we were able to derive the cosmological redshift, the process that not only required fewer assumptions, but also involved fewer steps.

## Conclusions

We introduce a general procedure to calculate the redshift parameter. By setting up a novel equation that establishes a relation between redshift and energy, we provide a fresh perspective on quantifying this phenomenon.

Knowing all the mechanisms that drive a change of energy in a given particle/photon, we are able to calculate its redshift. As we know, these mechanisms will be due to a difference in the frame of reference or to an actual loss of energy.

The introduction of an energy-dependent unified redshift relation not only enables a deeper understanding and interpretation of the redshift mechanisms but also facilitates the correlation between different interpretations. Furthermore, it allows the extension of redshift to encompass unexplored mechanisms, such as non-conservative effects or combinations of redshift mechanisms.

This procedure can be applied to a width diversity of systems, whether quantum, classical, or relativistic. The proposed interpretation, based on the energy of the particle, demonstrates that the implications of redshift phenomena extend far beyond the realm of astrophysics.

## Data Availability

All data generated or analyzed during this study are included in this published article.
